# AtEAU1 and AtEAU2, Two EAR Motif-Containing ABA Up-Regulated Novel Transcription Repressors Regulate ABA Response in *Arabidopsis*

**DOI:** 10.3390/ijms23169053

**Published:** 2022-08-13

**Authors:** Na Zhang, Siyu Chen, Adnan, Xutong Wang, Saddam Hussain, Yuxin Cheng, Yingying Li, Yuan Yuan, Chen Wang, Rao Lin, Huiyuan Zhang, Jiachen Wang, Tianya Wang, Shucai Wang

**Affiliations:** 1 Laboratory of Plant Molecular Genetics and Crop Gene Editing, School of Life Sciences, Linyi University, Linyi 276000, China; 2 Key Laboratory of Molecular Epigenetics of MOE, Northeast Normal University, Changchun 130024, China

**Keywords:** ABA, transcription factor, EAR motif-containing protein, AtEAUs, *Arabidopsis*

## Abstract

EAR (Ethylene-responsive element binding factor-associated Amphiphilic Repression) motif-containing transcription repressors have been shown to regulate plant growth and development, and plant responses to plant hormones and environmental stresses including biotic and abiotic stresses. However, the functions of most EAR-motif-containing proteins remain largely uncharacterized. The plant hormone abscisic acid (ABA) also plays important roles in regulating plant responses to abiotic stresses via activation/repression of ABA-responsive genes. We report here the identification and functional characterization of two ABA-responsive EAR motif-containing protein genes, *AtEAU1* (*Arabidopsis thaliana* *EAR motif-containing ABA* *Up-regulated 1*) and *AtEAU2*. Quantitative RT-PCR results show that the expressions of *AtEAU1* and *AtEAU2* were increased by ABA treatment, and were decreased in the ABA biosynthesis mutant *aba1-5*. Assays in transfected *Arabidopsis* protoplasts show that both AtEAU1 and AtEAU2 were specifically localized in the nucleus, and when recruited to the promoter region of the reporter gene by a fused DNA binding domain, repressed reporter gene expression. By using T-DNA insertion mutants and a gene-edited transgene-free mutant generated by CRISPR/Cas9 gene editing, we performed ABA sensitivity assays, and found that ABA sensitivity in the both *ateau1* and *ateau2* single mutants was increased in seedling greening assays. ABA sensitivity in the *ateau1* *ateau2* double mutants was also increased, but was largely similar to the *ateau1* single mutants. On the other hand, all the mutants showed a wild type response to ABA in root elongation assays. Quantitative RT-PCR results show that the expression level of *PYL4*, an ABA receptor gene was increased, whereas that of *ABI2*, a PP2C gene was decreased in the *ateau1* and *ateau1* single, and the *ateau1* *ateau2* double mutants. In summary, our results suggest that *AtEAU1* and *AtEAU2* are ABA-response genes, and AtEAU1 and AtEAU2 are novel EAR motif-containing transcription repressors that negatively regulate ABA responses in *Arabidopsis*, likely by regulating the expression of some ABA signaling key regulator genes.

## 1. Introduction

The plant hormone ABA (abscisic acid) regulates plant tolerance to abiotic stresses such as drought, salt, cold, and low oxygen, in most of the cases, via regulating the expression of downstream ABA response genes [1,2,3,4,5,6,7,8]. Under stress conditions, levels of ABA increase in plant cells, therefore ABA molecules are able to bind to the PYR1 (Pyrabactin Resistance 1)/PYL(PYR1-like)/RCAR (Regulatory Component of ABA Receptor) receptors [5,9,10,11,12,13]. The binding of ABA allows the PYR1/PYL/RCAR receptors to interact with the A-group PP2Cs (PROTEIN PHOSPHATASE 2C) phosphatases, which under normal conditions, bind to the SnRKs (NONFERMENTING 1 (SNF1)-RELATED PROTEIN KINASES) kinases and inhibit their activities [2,3,6,14]. Once released from A-group PP2Cs phosphatases, SnRKs kinases are self-activated, and then phosphorylate the ABF (ABA-responsive element-binding protein)/AREB(ABRE-binding factor)/ABI5 (ABA INSENSITIVE 5)-type bZIP (basic region leucine zipper) transcription factors. Phosphorylated ABF/AREB/ABI5 transcription factors are then able to regulate the expression of ABA-responsive genes, resulting in altered responses of plants to ABA and abiotic stresses [2,6,7,14,15,16,17].

In addition to the ABF/AREB/ABI5 transcription factors, some of the transcription factors from known transcription factor families encoded by ABA response genes are also able to regulate plant responses to ABA and abiotic stresses. For example, the expression of both B3 transcription factor gene *ABI3* and AP2 (APETALA2) transcription factor gene *ABI4* are induced by ABA, and ABI3 and ABI4 positively regulate ABA response in *Arabidopsis* [18,19]. On the other hand, expression of the bHLH transcription factor gene *bHLH129* is down-regulated by ABA, and bHLH129 negatively regulates ABA response in *Arabidopsis* [6]. In addition to transcription factors from known transcription factor families, some novel transcription factors have also been identified from unknown function ABA response genes. As an example, transcription repressor family genes *AITRs* (*ABA-induced transcription repressors*) were identified from unknown function ABA response genes, and AITRs negatively regulate ABA and abiotic tresses responses in *Arabidopsis* [6,7]. In particular, we found that some AITRs are EAR (ERF-associated amphiphilic repression) motif-containing proteins that contain a fully conserved LxLxL EAR motif [6].

The EAR motifs were first identified in the class II ERFs (ethylene-responsive factors) and some of the C2H2 family transcription factors that confer transcriptional repression activities [20], and then in other transcription repressors such as Aux/IAA proteins and ovate family proteins (OFPs) [21,22]. The EAR motifs were initially identified as a (L/F)DLN(L/F)xP amino acid signature [20]; further analysis of the EAR motifs in the class II ERFs, the C2H2 family proteins, and some other EAR motif-containing proteins have refined the amino acid signatures as DLNxxP and LxLxL, and transcriptional repression mediated by EAR motif-containing proteins has been considered as the main transcriptional repression form in plants [23].

Consistent with the importance of EAR motif-containing in regulating gene expression, accumulated evidence suggests that EAR motif-containing proteins are involved in the regulation of plant growth and development, and plant response to plant hormones and environmental stresses, including biotic and abiotic stresses, in most of the cases, via functioning as transcription repressors or recruiting co-repressors to repress the expression of the downstream target genes [22,23,24,25,26,27,28,29,30,31]. As examples, the transcription repressor OFP1 regulates cell elongation in *Arabidopsis* via directly repressing the expression of the plant hormone GA (Gibberellin) biosynthesis gene *GA20ox1* (*GA20-oxidase 1*) [22], KIX8 (KINASE-INDUCIBLE DOMAIN INTERACTING 8) and KIX9 represses *Arabidopsis* leaf growth via recruiting the co-repressor TOPLESS to form a repressor protein complex [32], whereas ERF7 regulates ABA and drought responses in *Arabidopsis* via interacting with the histone deacetylase HDA19, the protein kinase PKS3, and AtSin3, a homolog of a human global transcription corepressor [25].

Considering the importance of EAR motif-containing proteins, a genome-wide search of the LxLxL or DLNxxP EAR motif -containing proteins was performed in 71 different plant species, and more than 20,000 EAR motif-containing proteins were identified, with 556 encoded by 411 genes in *Arabidopsis*, but most of them are functionally uncharacterized [31]. On the hand, as the EAR motif-containing proteins show a high amino acid sequence diversity [31], it is very likely that some of the EAR motif-containing proteins may still remain unidentified. As an example, we found that some of the AITRs have a fully conserved LxLxL EAR motif [7], but none of them are identified in the genome-wide search of EAR motif-containing proteins [31]. Therefore we first tried to identify novel EAR motif-containing proteins that may involve in the regulation of ABA response by searching EAR motifs in proteins encoded by unknown function ABA-responsive genes from available transcriptome datasets, and we successfully identified SlEAD1 (*Solanum lycopersicum* EAR motif-containing ABA down-regulated 1) as a negative regulator of ABA response in tomato [33].

Here, by using an opposite strategy, i.e., identifying ABA response genes from unknown function EAR motif-containing protein genes [31], and searching for protein homologs, we identified AtEAU1 (*Arabidopsis thaliana* EAR motif-containing ABA Up-regulated 1) and AtEAU2 as novel regulators of ABA response in *Arabidopsis*.

## 2. Results

### 2.1. AtEAU1 and AtEAU2 Are Closely Related EAR Motif-Containing Proteins

A protein encoded by gene At1g78170 is among the identified EAR motif-containing proteins [31]. As a protein encoded by gene At1g22250 is closely related to the protein encoded by gene At1g78170, and the expression of these two genes is induced by ABA treatment (see next section for details), we named them *AtEAU1* (*Arabidopsis thaliana EAR motif-containing ABA* *Up-regulated 1*) and *AtEAU2*, respectively.

**Figure 1:**
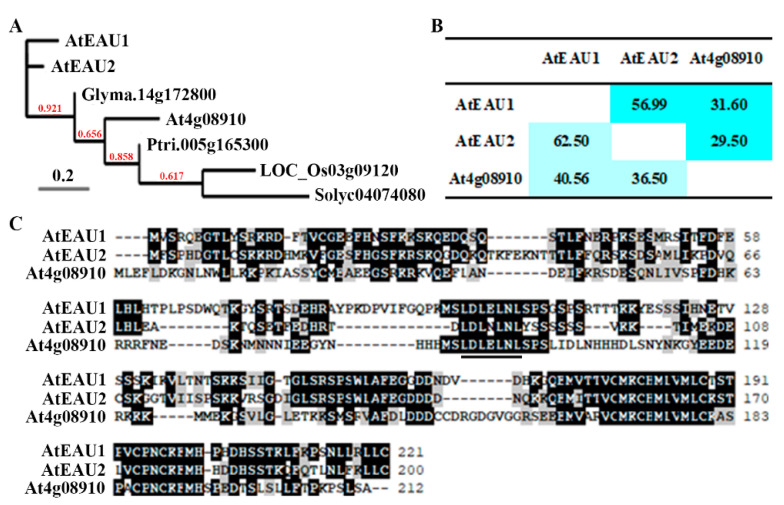
AtEAUs in *Arabidopsis*. (**A**) Phylogenetic analysis of AtEAU1, AtEAU2, and their closely related proteins. The full-length amino acid sequences of the AtEAU1 and AtEAU2 and their closely related proteins from *Arabidopsis* and other several different plants were obtained from phytozome (https://phytozome-next.jgi.doe.gov, accessed on 1 September 2016), and subjected to phylogenetic analysis by using the “One Click” mode with default settings on phylogeny (http://www.phylogeny.fr/simple_phylogeny.cgi, accessed on 1 September 2016). Numbers above the branches indicate the branch support values. (**B**) Amino acid identity and similarity of AtEAU1, AtEAU2 and At4g08910. The full-length amino acid sequences of AtEAU1, AtEAU2, and At4g08910 were used for SIAS (http://imed.med.ucm.es/Tools/sias.html, accessed on 1 September 2016) assay. The amino acid identity percentage was shaded in light blue, and the similarity percentage in blue. (**C**) Sequence alignment of AtEAUs and At4g08910. Black shades indicate the identical amino acids and gray shades indicate the similar amino acids. Underline indicates the LxLxL motif, one of the two known EAR motifs.

Protein homolog identification on Phytozome (https://phytozome-next.jgi.doe.gov, accessed on 1 September 2016) show that the next closely related protein to AtEAU1 and AtEAU2 is the protein encoded by gene At4g08910, which was also identified as an EAR motif-containing protein [31]. However, phylogenetic analysis shows that the most closely related protein identified from other plant species including soybean, tomato, rice and poplar are more closely related to At4g08910, and they formed a clade, whereas AtEAU1 and AtEAU2 formed another clade (Figure 1A).

Consistent with the phylogenetic tree, amino acid identity and similarity assays show that AtEAU1 and AtEAU2 shared ~57% amino acid identity and ~62% similarity, respectively, but sharing between At4g08910 and AtEAU1 or AtEAU2 was only about 30% and 40%, respectively (Figure 1B). On the other hand, even though At4g08910 shared relatively low amino acid identity and similarity with AtEAU1 or AtEAU2, it has a fully conserved LxLxL EAR motif presented in AtEAU1 or AtEAU2 (Figure 1C).

### 2.2. The Expression of AtEAU1 and AtEAU2 Are Up-Regulated by ABA and They Share Similar Expression Patern

Since AtEAU1 and AtEAU2 are closely related, but showed low amino acid identity and similarity with At4g08910, and are in a different clade than At4g08910 in the phylogenetic analysis (Figure 1), we characterized only AtEAU1 and AtEAU2 in this study.

**Figure 2:**
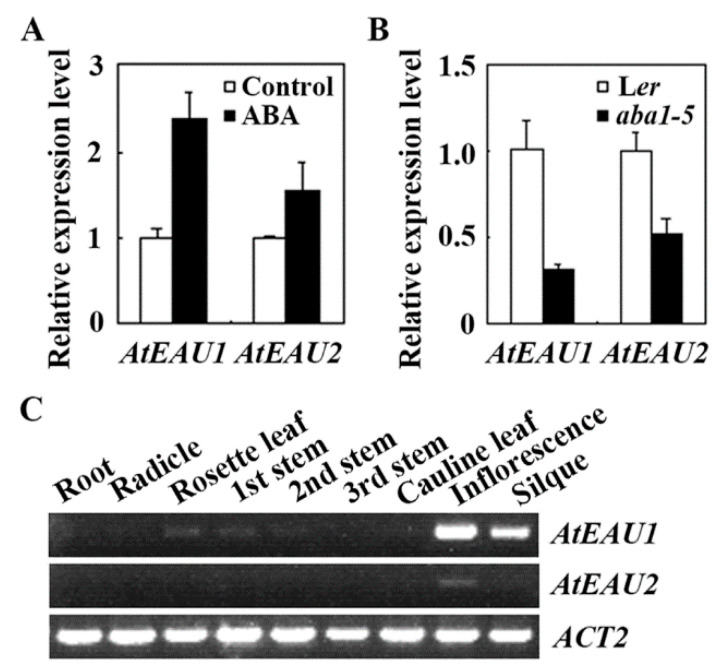
Expression of *AtEAUs* in seedlings treated with ABA and in different tissues and organs. (**A**) Expression of *AtEAU1* and *AtEAU2* in ABA-treated seedlings. Twelve-day-old seedlings of the Col wild type *Arabidopsis* were treated with 50 μM ABA for 4 h, RNA was then isolated and subjected to quantitative RT-PCR (qRT-PCR) to examine the expression of *AtEAU1* and *AtEAU2*. *ACT2* was used as an inner reference gene for qRT-PCR, and the expression levels of *AtEAU1* and *AtEAU2* in mock-treated control seedlings were set as 1. Data represent the mean ± SD of three replicates. (**B**) Expression of *AtEAU1* and *AtEAU2* in the *aba1-5* ABA biosynthesis mutant seedlings. RNA was isolated from 12-day-old seedlings of the *Ler* wild type and the *aba1-5* mutant, and qRT-PCR was used to examine the expression of *AtEAU1* and *AtEAU2*. *ACT2* was used as an inner reference gene for qRT-PCR, and expression of *AtEAU1* and *AtEAU2* in the *Ler* wild-type seedlings was set as 1. Data represent the mean ± SD of three replicates. (**C**) Expression pattern of *AtEAU1* and *AtEAU2*. Roots, radicle, rosette leaves, stems including 1st, 2nd, and 3rd stem from bottom to top of the inflorescence, cauline leaves, inflorescences, and siliques were collected from 5-week-old plants, RNA was then isolated and subjected to RT-PCR analysis to examine the expression of *AtEAU1* and *AtEAU2*. *ACT2* was used as a control for RT-PCR analysis.

Expression of *AtEAU1* and *AtEAU2* in response to exogenous ABA treatment was examined using the Col wild-type *Arabidopsis* seedlings. Seedlings of the Col wild type were treated with ABA, and RNA isolated was used for qRT-PCR analysis. As shown in Figure 2A, the expression levels of *AtEAU1* and *AtEAU2* increased about 2.5 and 1.5 fold, respectively, in seedlings treated with ABA compared with mock-treated seedlings. We also examined the expression of *AtEAU1* and *AtEAU2* in seedlings of the *aba1-5*, an ABA biosynthesis mutant in the *Ler* wild type background [34]. We found that the expression level of *AtEAU1* and *AtEAU2* decreased to about 1/3 and 1/2 of that in the *Ler* wild type seedlings, respectively (Figure 2B). These results suggest that *AtEAU1* and *AtEAU2* are ABA response genes.

We further examined the expression pattern of *AtEAU1* and *AtEAU2*. Different tissues and organs were collected from adult *Arabidopsis* plants, and RNA was isolated and used for RT-PCR analysis. As shown in Figure 2C, *AtEAU1* and *AtEAU2* shared a largely similar expression pattern, i.e., both have relative higher express levels in inflorescence and siliques. However, the expression of *AtEAU1* was also detectable in rosette leaves and stems, whereas *AtEAU2* was not, possibly due to its relatively low expression level (Figure 2C).

### 2.3. AtEAU1 and AtEAU2 Function as Transcription Repressors

As AtEAU1 has been identified as EAR motif-containing protein [31], and AtEAU2 also has a fully conserved LxLxL EAR motif, we thus examined if they may function as transcription repressors by using transfection assays in *Arabidopsis* protoplasts.

**Figure 3:**
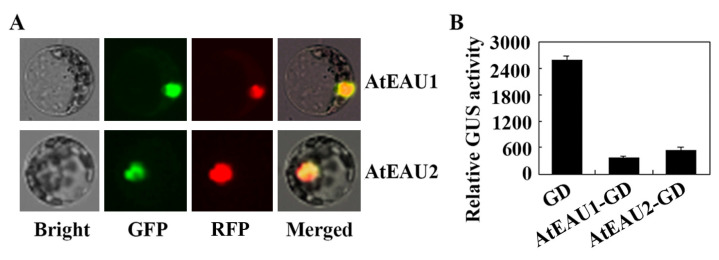
Protein subcellular localization and transcription activity of AtEAUs. (**A**) Protein subcellular localization of AtEAU1 and AtEAU2. Plasmid DNA of *AtEAU1-GFP* and *AtEAU2-GFP* was co-transfected with nucleus indicator *NLS-RFP* into protoplasts isolated from the Col wild-type *Arabidopsis*; transfected protoplasts were incubated for 20–22 h in darkness and then examined under a fluorescence microscope. Left panel: bright-field channel; middle panel: GFP channel; right panel: merged image. (**B**) Transcriptional activities of AtEAU1 and AtEAU2. Plasmid DNA of the *AtEAU1-GD* and *AtEAU2-GD* was cotransfected with a plasmid of the *LD-VP* activator gene, and the *LexA-Gal4:GUS* reporter gene into protoplasts isolated from the Col wild-type *Arabidopsis*, and the transfected protoplasts were incubated for 20–22 h in darkness before GUS activity was assayed. Cotransfection of the *GD* plasmid DNA was used as a control. Data represent the mean ± SD of three replicates.

We first examined the protein subcellular localization of AtEAU1 and AtEAU2. Plasmids of the *AtEAU1-GFP* and *AtEAU2-GFP* constructs were transfected into *Arabidopsis* protoplasts isolated from leaves of 3–4 weeks old Col wild type plants, and GFP fluorescence in the protoplasts was observed under a confocal microscope. As shown in Figure 3A, GFP fluorescence for both AEAU1-GFP and AtEAU2-GFP was observed only in the nucleus, suggesting that AEAU1 and AtEAU2 are nucleus proteins.

We then examined if AtEAU1 and AtEAU2 may able to repress reporter gene expression in transfected protoplasts when recruited to the promoter of the reporter gene by a fused DNA binding domain. Plasmids of the reporter construct *LexA*-*Gal4:GUS* and the transcriptional activator construct *LD-VP* were co-transfected with the effect construct *AtEAU1-GD* or *AtEAU2-GD* into *Arabidopsis* protoplasts. Co-transfection of effector construct *GD* was used as a control. As the transcriptional activator LD-VP is able to bind to the *LexA* promoter via the fused LD domain and activate the expression of the *LexA*-*Gal4:GUS* reporter gene, whereas the AtEAU1 or AtEAU2 proteins are able to bind to the *Gal4* promoter via the fused GD domain, expression levels of the reporter gene will be decreased if AtEAU1 and AtEAU2 function as transcription repressors. As shown in Figure 3B, compared with cotransfection of *GD*, GUS activity was dramatically reduced when *AtEAU1-GD* or *AtEAU2-GD* was cotransfected, suggesting that AtEAU1 and AtEAU2 are transcription repressors.

### 2.4. Generation of Single and Double Mutants for Gene AtEAU1 and AtEAU2

**Figure 4:**
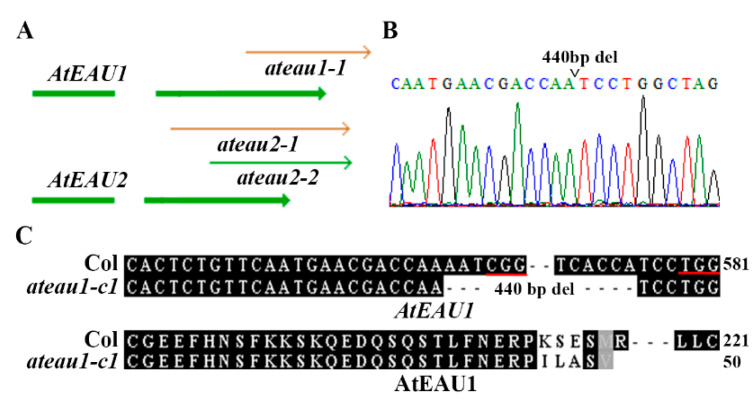
Isolation of the T-DNA insertion mutants for *AtEAUs* and generation of the gene-edited mutant for *AtEAU1*. (**A**) The T-DNA insertion position of the *ateau1-1*, *ateau2-1*, and *ateau2-2* single mutants. Seeds of the T-DNA lines SAIL_197_H05, SAIL_642_C12, and SAIL_1242_E02 were obtained from ABRC, and homozygous mutants were identified by genotyping. (**B**) Gene editing status of *AtEAU1* in the *ateau1-c1* mutant. DNA was isolated from leaves of the T2 transgene-free plants, and subjected to PCR amplification of the genome sequence of *AtEAU1*. The PCR products were recovered and sequenced, and the sequencing results obtained were compared with *AtEAU1* genome sequence to check the editing status. Open arrowhead indicates the position where the 440 bp fragment was deleted. (**C**) Alignment of the nucleotide sequences (up panel) of *AtEAU1* and amino acid sequences (low panel) of AtEAU1 in the Col wild type and the *ateau1-c1* mutant. In the nucleotide sequence alignment, numbers beside sequences indicate the relative position of the nucleotides to the start codon, and underlines indicate the PAM sites. In the amino acid alignment, *AtEAU1* sequences in the mutant were subjected to ORFfinder (https://www.ncbi.nlm.nih.gov/orffinder/, accessed on 1 September 2016) for ORF analysis, and the predicted amino acid sequences were aligned with AtEAU1 amino acid sequence. Numbers beside the sequences indicate the total amino acids in the corresponding proteins.

In order to examine if AtEAU1 and AtEAU2 may involve in the regulation of ABA response in *Arabidopsis*, we isolated/generated single and double mutants for *AtEAU1* and *AtEAU2*. Since there is 1 T-DNA insertion line available for gene *AtEAU1*, and 2 are available for *AtEAU2* from ABRC, we obtained the seeds and isolated single homozygous mutants *ateau1-1* (SAIL_197_H05), *ateau2-1* (SAIL_642_C12), and *ateau2-2* (SAIL_1242_E02) (Figure 4A). By crossing *ateau1-1* and *ateau2-1* mutants and examining F2 plants, we obtained *ateau1 ateau2* double mutants.

Considering that only one T-DNA insertion mutant was obtained for *AtEAU1*, we decided to generate an additional mutant by using CRISPR/Cas9 gene-editing of *AtEAU1*. Two different target sequences were selected and used to generate CRISPR/Cas9 construct. By transforming the Col wild-type *Arabidopsis*, examining gene editing status in T1 transgenic plants, and isolating Cas9-free homozygous mutants from T2 plants, we successfully obtained a transgene-free homozygous fragment deletion mutant, namely *ateau1-c1*. Sequencing results show that there is a 440bp fragment deletion in the *ateau1-c1* mutant (Figure 4B). The deletion led to substitutions of a few amino acids and a premature stop in *AtEUA1*, resulting in a short amino acid sequence of AtEAU1 with only 50 amino acids in the *ateau1-c1* mutant (Figure 4C).

### 2.5. ABA Sensitivity Is Increased in the Single and Double Mutants of AtEAU1 and AtEAU2

By using the mutants obtained, we examined the role of AtEAU1 and AtEAU2 in regulating of ABA response in *Arabidopsis* using seedling greening assay. Seeds of the Col wild type, the *ateau1* and *ateau2* single and the *ateau1 ateau2* double mutants were plated on 1/2 MS plates at the presence of 1 µM ABA or solvent alone as a control, and seedlings with green cotyledons were counted 14 days after the transfer of the plates into the growth room. The percentage of green cotyledons was then calculated.

**Figure 5:**
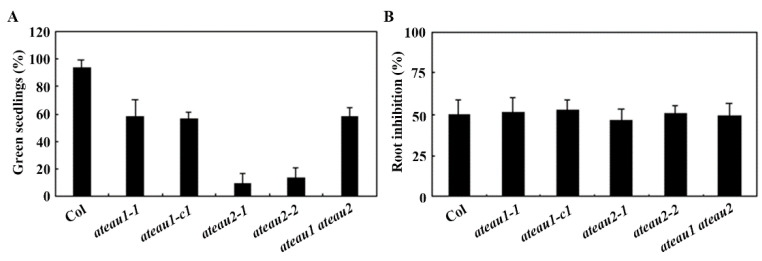
Loss-of-function of *AtEAUs* enhanced ABA response in *Arabidopsis*. (**A**) Cotyledon greening of the Col wild type *Arabidopsis*, the *ateau1* and *ateau2* single, and the *ateau1 ateau2* double mutants in response to ABA treatment. Sterilized seeds were sown on 1/2 MS plates containing 1 µM ABA or solvent control, respectively. The plates were kept for 2 days at 4 °C in darkness, and then transferred to a growth room. Fourteen days after the transfer, seedlings with green cotyledons were counted and the percentage of green cotyledons was then calculated. Data represent the mean ± SD of three replicates. (**B**) Root elongation of the Col wild type, the *ateau1* and *ateau2* single, and the *ateau1 ateau2* double mutants in response to ABA treatment. Sterilized seeds were sown on 1/2 MS plates, kept for 2 days at 4 °C in darkness, then transferred to a growth room and grown vertically for 2 days. Seedlings with similar root length were selected and transferred to plates containing 5 µM ABA or solvent control, respectively. New elongated root length was measured, and percentage of inhibition was then calculated. Data represent the mean ± SD of 10 seedlings.

We found that, on the control plates, seedlings for all the plants turned to green, however, on the ABA-containing plates, the green seeding rate for the Col wild type was nearly 95%, whereas that for both the *ateau1-1* and *ateau1-c1* single mutants was about 60%, and that for the *ateau2* single mutants was less than 20% (Figure 5A). To our surprise, we found that the green seeding rate for the *ateau1 ateau2* double mutants was about 60%, similar to the *ateau1-1* and *ateau1-c1* single mutants (Figure 5A).

We also examined response of the mutants to ABA by using root elongation inhibition assays. Sterilized seeds of the Col wild type, the *ateau1* and *ateau2* single and the *ateau1 ateau2* double mutants were plated on 1/2 MS plates and grown vertically for 2 days, then seedlings with root at the same length were transferred to 1/2 MS plates at the presence or absence of 5 µM ABA and grown for 9 additional days, then new elongated root length was measured and the percentage of inhibition was calculated. We found that root elongation of all the seedlings, including the Col wild type, the *ateau1* and *ateau2* single, and the *ateau1 ateau2* double mutants was inhibited by about 50% (Figure 5B).

### 2.6. Expression Levels of Some ABA Signaling Genes Are Altered in the Single and Double Mutants of AtEAU1 and AtEAU2

**Figure 6:**
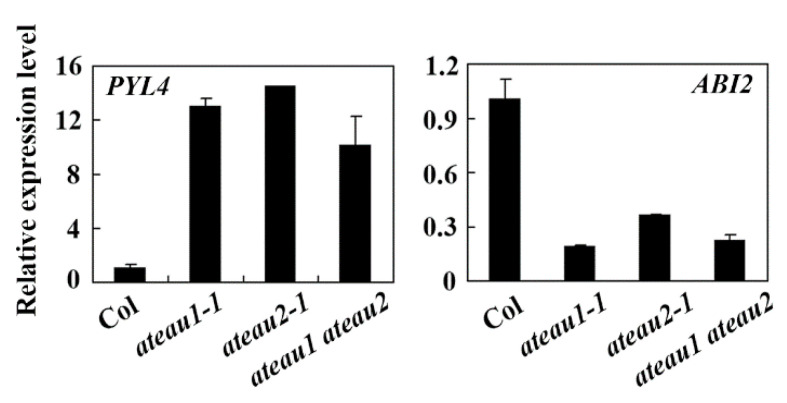
Expression of *PYL4* and *ABI2* in mutants of *AtEAUs*. RNA was isolated from 12-day-old seedlings of the Col wild type, the *ateau1-1, ateau2-1* single, and the *ateau1 ateau2* double mutants, and used for qRT-PCR analysis. Expression of *ACT2* was used as an inner reference gene, and expression of *PYL4* and *ABI2* in the seedlings of the Col wild-type plants was set as 1. Data represent the mean ± SD of three replicates.

Having shown that *AtEAU1* and *AtEAU2* are ABA response genes, and ABA sensitivity was increased in the *ateau1* and *ateau2* single and the *ateau1 ateau2* double mutants, we wanted to further examine how AtEAU1 and AtEAU2 may regulate plant response to ABA. We examined the expression of ABA single key regulator genes in seedlings of the Col wild type, the *ateau1-1* and *ateau2-1* single, and the *ateau1 ateau2* double mutants, and found that the expression level of the ABA receptor gene *PYL4* was increased, but the PP2C gene *ABI2* was decreased in the seedlings of the *ateau1-1* and *ateau2-1* single and the *ateau1 ateau2* double mutants when compared with that in the seedlings of the Col wild type plants (Figure 6).

## 3. Discussion

EAR motif-containing-protein mediated transcriptional repression has been considered to be the main transcriptional repression form in plants [23], consistent with this, EAR motif-containing proteins are involved in the regulation of plant growth and development, as well as plant response to hormones and environmental stresses [22,23,24,25,26,28,29,30,31]. So far it has also been shown that EAR motif-containing proteins are involved in the regulation of hormone responses including ethylene, jasmonate, auxin, and strigolactone [20,23,26,27,28]. Some of the EAR motif-containing proteins, including ERF7, AITRs, and SlEAD1 have been shown to regulate ABA response in plants [6,25,33]. We provide evidence here that AtEAU1 and AtEAU2 are ABA responsive novel EAR motif-containing transcription repressors that regulating ABA response in *Arabidopsis*.

First, AtEAU1 has previously reported to be an EAR motif-containing protein [31], and our bioinformatics analysis identified AtEAU2 as its closely related EAR motif-containing protein (Figure 1). Second, we found that the expression levels of *AtEAU1* and *AtEAU2* were increased in response to exogenously ABA treatment, but decreased in the ABA biosynthesis mutant *aba1-5* (Figure 2). Third, consistent with the fact that AtEAU1 and AtEAU2 are closely related and both have a fully conserved LxLxL EAR motif (Figure 1), both AtEAU1 and AtEAU2 were specifically localized in the nucleus in *Arabidopsis* protoplast assays, and functioned as transcription repressors (Figure 3). Last but not least, ABA sensitivity was increased in the *ateau1* and *ateau2* single mutants in green seedling assays (Figure 5). These results show that *AtEAU1* and *AtEAU2* are ABA responsive EAR motif-containing protein genes, and AtEAU1 and AtEAU2 function as transcription repressors to regulate ABA response in *Arabidopsis*. However, in root elongation assays, no difference was observed between the seedlings of the Col wild type and the mutants, suggesting the AtEAU1 and AtEAU2 may not regulate ABA sensitivity at root elongation stage.

Considering that both *AtEAU1* and *AtEAU2* are ABA response genes (Figure 2), and AtEAU1 and AtEAU2 shared higher amino acid identity and similarity (Figure 1), and repressed reporter gene expression in transfected protoplasts (Figure 3), we expect that they may have redundant functions, therefore we generated *ateau1 ateau2* double mutant, and tested whether that was the case. However, we found that the *ateau2* single mutants display greater sensitivity to ABA than the *ateau1* single mutants, but sensitivity in the *ateau1 ateau2* double mutant is largely similar to the *ateau1* single mutants (Figure 5), and expression level changes of *PYL4* and *ABI2* in the *ateau1 ateau2* double mutant are also largely similar to those in the *ateau1* single mutants (Figure 6). These results suggest that AtEAU1 and AtEAU2 may function sequentially to regulate ABA response in *Arabidopsis*, and AtEAU1 may function down stream of AtEAU2. However, more experiments are required to examine if that is the case.

Consistent with the key roles played by ABA in regulating plant abiotic stress responses via regulating the expression of downstream ABA response genes, changes in the expression of ABA-signaling key regulator genes, including the PYR1/PYL/RCAR receptor genes, the PP2Cs phosphatase genes, the SnRKs kinase genes, and ABF/AREB/ABI5-type bZIP transcription factor genes all affected plant responses to abiotic stresses [1,2,3,4,5,6,7]. On the other hand, changes in the expression of some downstream ABA response genes also affected plant abiotic stress response, for example, the R2R3 MYB transcription gene *MYB44*, the heat shock factor gene *HSFA6b*, and the bHLH transcription factor gene *bHLH112* are all ABA responsive genes involved in the regulation of plant responses to abiotic stresses [35,36,37]. Consistent with the importance of EAR motif-containing proteins in regulating transcriptional repression in plants [23], some EAR motif-containing proteins such as ERF7, AITRs, and SlEAD1 have been shown to be involved in regulating ABA responses [6,25,33]. So far we have used two different strategies to identify EAR motif-containing proteins; i.e., searching EAR motifs in proteins encoded by ABA-responsive genes with unknown functions, and identifying ABA response genes from unknown function EAR motif-containing protein genes [31]. By using the first strategy, we have successfully identified SlEAD1 as a novel negative regulator of ABA response in tomato [33], and here by using the second strategy, we identified AtEAU1 and AtEAU2 as novel regulators of ABA response in *Arabidopsis*, indicating that both strategies are practical. Since most of the EAR motif-containing proteins that have been identified are functionally uncharacterized, and some of the EAR motif-containing proteins may have not been identified due to their sequence diversity [31,33], more EAR motif-containing ABA response regulator should be identified by using the two strategies mentioned above, and some of the genes may be used for plant breeding to improve plant abiotic stress tolerance.

Nevertheless, we identified the EAR motif-containing protein genes *AtEAU1* and *AtEAU2* as ABA responsive genes, we showed that AtEAU1 and AtEAU2 function as transcription repressors, and we found that AtEAU1 and AtEAU2 negatively regulate ABA responses in *Arabidopsis*, possibly by regulating the expression of the ABA signaling key regulator genes *PYL4* and *ABI2*.

## 4. Materials and Methods

### 4.1. Bioinformatics Analysis

Homologs of AtEAU1 (At1g78170) in *Arabidopsis*, soybean, rice, tomato, and poplar were identified on Phytozome (https://phytozome-next.jgi.doe.gov, accessed on 1 September 2016) under the term Protein Homologs, and homologs were then used to search on Phytozome again to make sure their homolog protein is AtEUA1. The full-length amino acid sequences of AtEAU1 and its protein homologs were used for phylogenetic analysis on Phylogeny (http://www.phylogeny.fr/simple_phylogeny.cgi, accessed on 1 September 2016) by using the “One Click” mode with default settings, and for sequence alignment using Bioedit 7.2. Amino acid sequence identity and similarity assays of AtEAU1, AtEAU2 and At4g08910 were analyzed on SIAS (http://imed.med.ucm.es/Tools/sias.html, accessed on 1 September 2016).

### 4.2. Plant Materials and Growth Conditions

The Columbia-0 (Col) ecotype *Arabidopsis* (*Arabidopsis thaliana*) was used as a wild-type for plant transformation, protoplast isolation, and as a control for ABA response analysis. Seeds of SAIL_197_H05, a T-DNA insertion line for *AtEAU1*, and SAIL_642_C12 and SAIL_1242_E02, T-DNA insertion lines for *AtEAU2* (At1g22250) obtained from the ABRC (Arabidopsis Biological Resource Center) are all in Col background, and were used to isolate the *ateau1-1* and *ateau2-1* and *ateau2-2* mutants, respectively. The *ateau1* *ateau2-1* double mutant was generated by crossing *ateau1-1* and *ateau2-1* single mutants, and identifying homozygous double mutants in the F2 generation. The *Ler* wild type and the *aba1-5* mutant in *Ler* background [34] were used to examine the expression of *AtEAUs*.

To generate plants for protoplast isolation and plant transformation, seeds of the Col wild type were sown directly into soil pots, germinated and grown in a growth room. To generate seedlings for RNA isolation and ABA response assays, seeds of the Col wild-type, the *ateau1* and *ateau2* single and *ateau1 ateau2* double mutants, the *Ler* wild type, and the *aba1-5* mutant were sterilized for 10 min with 25% (*v*/*v*) bleach, washed with sterilized water four times, and plated on 0.6% (*w*/*v*) phytoagar (PlantMedia) solidified 1/2 MS (Murashige and Skoog) plates with vitamins (Plant Media) and 1% (*w*/*v*) sucrose. The plates were kept in darkness at 4 °C for 2 days, and then transferred to a growth room. The growth conditions in the growth room were set at 22 °C, and a 16 h light/8 h dark long-day condition with photon density at ~120 μmol m^−2^ s^−1^.

### 4.3. RNA Isolation and RT-PCR

To examine the expression changes of *AtEAU1* and *AtEAU2* in response to ABA treatment, 12-day-old seedlings of the Col wild-type were treated for 4 h with 50 µM ABA or solvent methanol, and then RNA was isolated. To examine the expression levels of *AtEAU1* and *AtEAU2* in the *aba1-5* mutant seedlings, RNA was isolated from 12-day-old seedlings of the *Ler* wild type and the *aba1-5* mutant. To examine the expression of ABA signaling genes in the *ateau1-1* and *ateau2-1* single and *ateau1 ateau2* double mutants, RNA was isolated from seedlings of 12-day-old Col wild-type, *ateau1-1* and *ateau2-1* single and *ateau1 ateau2* double mutants. To examine the expression pattern of *AtEAUs*, roots, radicle, rosette leaves, first stems, second stems, third stems, cauline leaves, inflorescences, and siliques were collected from 5-week-old plants, and used for RNA isolation.

Total RNA isolated was used for cDNA synthesis as described previously [38], and then used to examine the expression of *AtEAUs* and ABA signaling genes by using RT-PCR/qRT-PCR. The primers used for *AtEAU1* were 5′-TTCATGCACCCACACGATCA-3′ and 5′-AGGTTCTTCTCTACAAAAAGCCTAA-3′; for *AtEAU2* they were 5′-TCAAAAGGAGCAAACAAGGAGA-3′ and 5′-GGCTTTATCAACATGGCGCT-3′. *ACT2* (*ACTIN2*) was used as a control gene for RT-PCR and an inner reference gene for qRT-PCR. The primers for *PYL4*, *ABI2*, and *ACT2* were as reported previously [39,40,41].

### 4.4. Construsts

The reporter gene *LexA-Gal4:GUS*, the control effector gene *GD*, the transcription activator gene *LD-VP*, and the nucleus indicator gene *NLS-RFP* used for *Arabidopsis* protoplast transfection were as described previously [21,22,42]. To generate *GD* and *GFP*-fused *AtEAU1* and *AtEAU2* constructs used for *Arabidopsis* protoplast transfection, the full-length ORF (open reading frame) sequences of *AtEAU1* and *AtEAU2* were RT-PCR amplified by using RNA isolated from 12-day-old seedlings of the Col wild type, digested with proper enzymes, and cloned under the control of the CaMV *35S* promoter, and placed in frame with a C-terminal GD or GFP tag into *pUC19* vector [21,22]. To generate CRISPR/Cas9 construct for gene editing of *AtEAU1*, exon sequences of *AtEAU1* were used for CRISPRscan (http://www.crisprscan.org/?page=sequence, accessed on 1 September 2016) to identify potential target sequences. Selected target sequences were evaluated for potential offtarget on Cas-OFFinder (http://www.rgenome.net/cas-offinder/, accessed on 1 September 2016). Two specific target sequences, i.e., 5′-TTCAATGAACGACCAAAAT (CGG)-3′ and 5′-GACTAAGTCGATCACCATCC (TGG)-3′, were selected. The target sequences were cloned into the *pHEE401E* vector as described previously in [43]. The primers used to generate the CRISPR/-Cas9 construct for editing *AtEAU1* were DT1-BsF (*AtEAU1*), 5′-ATATATGGTCTCGATTGTTCAATGAACGACCAAAAT GTT-3′, DT1-F0 (*AtEAU1*), 5′-TGTTCAATGAACGACCAAAATGTTTTAGAGCTAGAAATAGC-3′, DT2-R0 (*AtEAU1*), 5′-AACGGATGGTGATCGACTTAGTCAATCTC TTAGTCGACTCTAC-3′, and DT2-BsR (*AtEAU1*), 5′-ATTATTGGTCTCGAAACGGATGGTGATCGACTTAGTC-3′. The U626-IDF and U629-IDR primers used for colony PCR and sequencing of the CRISPR/Cas9 constructs generated were described previously [44].

### 4.5. Plant Transformation and Transgenic Plants Selection

About 5-week-old Col wild-type plants with several mature flowers were transformed with the CRISPR/Cas9 constructs to generate the *ateau1-c1* single mutant by using the floral dip method [45]. Transgenic plants generated were selected by plating the T1 seeds collected on 1/2 MS plates with 30 μg/mL Hygromycin and 100 μg/mL Carbenicillin. Gene editing status in the T1 plants was examined by PCR amplification and sequencing the genomic sequence of *AtEAU1*. T2 seeds were collected from gene-edited T1 plants, germinated directly in soil pots, and used to identify Cas9-free homozygous mutant as described in the next section.

### 4.6. DNA Isolation and PCR

To examine gene editing status of *AtEAU1*, DNA was isolated from leaves of the T1 transgenic plants or the Cas9-free T2 plants, and used for PCR amplification of the genomic sequences of *AtEAU1*. To isolate transgene-free mutants, DNA was isolated from leaves of the T2 offspring of the gene-edited T1 plants, and subjected to PCR amplification of the *Cas9* gene fragment by using the primers described previously [44].

### 4.7. Plasmid DNA Isolation, Protoplast Isolation and Transfection

Plasmid DNA of the reporter and effector genes was isolated by using the Gold Hi Endo Free Plasmid Maxi Kit (CWBIO) according to the manufacturers’ instructions. Protoplasts were isolated from rosette leaves collected from 3–4-week-old Col wild-type plants, and the isolated protoplasts were transfected as described previously [6,38,46,47]. For subcellular location assays, plasmid DNA of *AtEAU1-GFP* and *AtEAU2-GFP* were transfected into protoplasts. For transcriptional activity assays, plasmid DNA of the reporter gene *LexA-Gal4:GUS*, the activator gene *LD-VP*, and the effector genes *GD*, *AtEAU1-GD*, or *AtEAU2-GD* were cotransformed into protoplasts. The transfected protoplasts were incubated in darkness at room temperature for 20–22 h, then GFP fluorescence was observed under an Olympus FV1000 confocal microscope, and GUS activities were measured using a Synergy^TM^ HT microplate reader.

### 4.8. ABA Sensitivity Assays

Assays of ABA-inhibited cotyledon greening and root elongation were performed as described previously [6,48,49,50]. For cotyledon greening assays, green seedlings on plates containing 1 µm ABA or solvent alone were counted 14 days after the plates were transferred to the growth room. For root elongation assays, 2-day-old seedlings grown on vertically placed plates were transferred to plates containing 5 µm ABA or solvent as a control; the length of new elongated roots was measured 9 days after the transfer. All the experiments were repeated at least three times.

## Data Availability

All data were obtained were presented in this article.
